# Electrocardiogram Embeddings Beyond Chronological Age for Prediagnostic Cognitive Impairment and Dementia: Development and External Validation Study

**DOI:** 10.2196/108392

**Published:** 2026-10-23

**Authors:** Yujeong Kim, Dong Won Kim, Yu Rang Park, Woo Jung Kim, Jaewoong Kim, Yujee Chang, Heeyeon Kwon, Dukyong Yoon

**Affiliations:** 1 Department of Biomedical Systems Informatics Yonsei University College of Medicine Seoul Republic of Korea; 2 Yonsei Institute for Digital Health Yonsei University Seoul Republic of Korea; 3 Department of Psychiatry Yongin Severance Hospital Yonsei University College of Medicine Yongin, Gyeonggi-do Republic of Korea; 4 Yonsei BEYOND Lab Yongin, Gyeonggi-do Republic of Korea; 5 Institute for Medical Engineering and Science Massachusetts Institute of Technology Cambridge, MA United States; 6 Center for Digital Health Yongin Severance Hospital Yonsei University Health System Yongin, Gyeonggi-do Republic of Korea

**Keywords:** dementia, cognitive impairment, electrocardiogram, deep learning, embeddings, age-complementary

## Abstract

**Background:**

Dementia is a growing health burden worldwide; however, scalable, low-cost strategies for early risk identification are lacking. Because cardiac and cognitive aging are closely associated, a routinely collected 12-lead electrocardiogram (ECG), interpreted using foundation model embeddings, is a promising and accessible candidate marker. However, it is unclear whether such embeddings carry predictive information independent of chronological age—the dominant risk factor for dementia—and whether the underlying changes follow a detectable prediagnostic trajectory.

**Objective:**

This study aimed to investigate whether foundation model embeddings of routine ECGs carry information that distinguishes patients later diagnosed with cognitive impairment or dementia from matched controls, beyond chronological age, and, if so, whether the underlying ECG changes intensify as diagnosis approaches.

**Methods:**

In a retrospective two-site Korean cohort (Severance Hospital, development n=52,355; Yongin Severance Hospital, external n=6500), embeddings from the knowledge-enhanced diagnosis foundation model supported an ECG-only classifier that discriminated patients diagnosed with cognitive impairment or dementia within the following 10 years from matched controls, using only ECGs interpreted as normal sinus rhythm. We compared an age-only model with a late fusion of age and ECG-derived probability and tested whether age-residualized embedding is predictive as direct evidence of age-independent information. Discrimination was summarized by the area under the receiver operating characteristic curve (AUROC) with 95% bootstrap CIs, and DeLong tests and net reclassification improvement (NRI) were used for between-model comparisons. To trace the prediagnostic time course, we analyzed ECG phenotype labels across four windows before diagnosis in an exact-age-matched case-control set.

**Results:**

Chronological age strongly discriminated cases from controls in both cohorts (external AUROC 0.809, 95% CI 0.799-0.819). Adding ECG-derived probability improved discrimination, calibration, and net benefit over age alone (external AUROC 0.825, 95% CI 0.816-0.834; DeLong *P*<.001; positive NRI). Critically, even after removing the age-predictable component, age-residualized embedding retained modest but reproducible discrimination in both internal (AUROC 0.590, 95% CI 0.581-0.599) and external (AUROC 0.579, 95% CI 0.567-0.592) validation (both *P*<.001), indicating dementia-relevant information that was not explained by chronological age. Of the 105 ECG labels, 19 (18.1%) showed a case-control difference that widened significantly toward diagnosis, dominated by nonspecific ST segment and T wave changes, acute ischemic patterns (acute myocardial infarction), and atrial and junctional arrhythmias (atrial flutter, ectopic atrial, and accelerated junctional rhythms), with divergence emerging mainly in the proximal (1-3-year) and imminent (0-1-year) windows.

**Conclusions:**

Routine ECG foundation model embeddings encode aging-related electrophysiological information that is complementary to and not fully explained by chronological age and intensifies as diagnosis approaches, with concurrent shifts toward ischemic and arrhythmic ECG patterns whose mechanism cannot be determined from these data. These findings suggest that routine ECG is a low-cost, age-complementary triage signal for cognitive impairment and dementia screening, rather than a stand-alone diagnostic test.

## Introduction

### Background

Dementia is a progressive syndrome characterized by deteriorating memory, cognition, and daily functioning. It affects tens of millions of people worldwide and imposes a substantial and rising health and economic burden [1,2]. Early identification of at-risk individuals has become increasingly consequential: disease-modifying antiamyloid therapies now slow clinical decline in early symptomatic Alzheimer disease, and their benefit depends on starting treatment before substantial neurodegeneration occurs. Therefore, early detection is crucial. However, no scalable, low-cost approach for identifying at-risk individuals is currently available because ascertainment relies on specialized cognitive testing (eg, Mini-Mental State Examination and Alzheimer’s Disease Assessment Scale) and neuroimaging (magnetic resonance imaging and positron emission tomography), which are better suited for diagnostic confirmation than population-level screening [3-6]. A practical alternative is to draw on data already acquired on the scale during routine clinical care.

### Prior Work

Cardiac and cognitive function decline in parallel with aging, sharing substrates that include neurohormonal dysregulation, chronic low-grade inflammation, and cellular senescence [7]. Studies on biological aging have shown that organ-specific aging can be quantified independently of chronological age and predicts long-term outcomes more accurately than age alone [8,9]. Together, these observations suggest that cardiac measurements may carry information about future cognitive trajectories. Consistent with the heart-brain axis, cardiovascular conditions, such as atrial fibrillation and heart failure, are associated with an elevated risk of dementia [10-12], and specific electrocardiographic abnormalities (eg, prolonged QT intervals, ST segment deviation, T wave abnormalities, and reduced heart rate variability) are more prevalent among individuals with dementia or mild cognitive impairment [13-18]. The 12-lead electrocardiogram (ECG) is noninvasive and widely available and may therefore provide an accessible measure of the cardiac and autonomic aging processes that accompany neurodegeneration.

However, conventional ECG interpretation is confined to a predefined set of visually recognizable features and may not capture the full physiological information contained in the waveform. Foundation models for ECG, such as the knowledge-enhanced diagnosis (KED) model, encode raw waveforms into compact embeddings that capture morphological and rhythmic characteristics beyond those identifiable by visual inspection alone [19,20]. Deep learning models have been shown to infer latent physiological information, including biological age, from the ECG [21,22]. Whether such embeddings carry dementia-relevant information not fully explained by chronological age and whether the underlying ECG changes evolve in the years preceding diagnosis have not been established in large-scale cohorts.

### Study Objectives

We tested two hypotheses in two cohorts with external validation, exclusively using ECGs clinically interpreted as normal sinus rhythms. First, we hypothesized that ECG foundation model embeddings carry information for early identification of cognitive impairment and dementia that adds to chronological age; as direct evidence of age-independent information, we tested whether an age-residualized embedding (each dimension orthogonalized to age) remains predictive. Second, we hypothesized that if this information reflects a genuine disease process, it should be tied to disease progression so that as diagnosis approaches, ECG changes become more pronounced and the divergence from controls widens. We tested this by characterizing the prediagnostic temporal trajectory of ECG features on an age-matched case-control set.

## Methods

### Study Design and Data Sources

This retrospective multisite study used data from two affiliated hospitals in South Korea: Severance Hospital (Severance) and Yongin Severance Hospital (YSH). The dataset comprised 12-lead, 10-second ECG waveforms and structured electronic medical records (EMRs) spanning November 1, 2005, to December 31, 2022. All ECGs were recorded using the GE HealthCare MUSE system. Severance ECGs were accessed through the Severance Data Portal. YSH ECGs were extracted from institutional servers, with clinical data retrieved from the Severance Clinical Research Analysis Portal for Anonymous. The structured EMRs provided demographic data (age and sex), comorbidity history, and lifestyle factors, which were used to characterize the cohorts.

### Participant Selection and Research Tasks

We considered all patients aged ≥18 years who had visited either hospital between November 2005 and December 2022 and had undergone at least one ECG. The case group included patients with a confirmed diagnosis of cognitive impairment or dementia (*International Statistical Classification of Diseases and Related Health Problems, Tenth Revision* [ICD-10] codes F00-F03, F06.7, F10.7, and G30-G31) and at least one ECG within 10 years before the initial diagnosis. Controls were drawn from the same source population as cases—patients aged ≥18 years with at least one ECG recorded at the participating hospitals between November 1, 2005, and December 31, 2022—and were defined as individuals with no ICD-10 code for cognitive impairment or dementia recorded at any point during the entire study period. No minimum duration of observation after the index ECG was required, and diagnoses established outside the participating institutions could not be ascertained from these data. To ensure that any predictive signal would arise from ECGs a clinician would read as unremarkable rather than from overt rhythm disturbance, patients with a history of arrhythmia (ICD-10 codes I44-I49) or cardiovascular surgery were excluded, ECGs flagged as abnormal or pacemaker-generated were removed, and only ECGs annotated as normal sinus rhythm were retained. This restriction was chosen so that the analysis would test whether ECGs without overt abnormalities, which currently provide no actionable information about cognitive risk, contain information associated with future cognitive impairment and dementia, rather than reflecting established associations between overt cardiac disease and dementia.

In Severance, a total of 40,312 cases and 2,844,812 controls were identified, of which 25,580 (63.5%) cases and 1,091,667 (38.4%) controls, respectively, had undergone at least one ECG. After exclusion and restriction to normal sinus rhythm, 10,471 (40.9%) cases (26,837 ECGs) and 41,884 (3.8%) controls (58,764 ECGs), matched in a 1:4 (case-control) ratio by propensity score for each individual’s median age at ECG acquisition and sex, were retained. Similarly, in the YSH, of a total of 7453 cases and 306,791 controls identified, 3584 (48.1%) cases and 116,051 (37.8%) controls, respectively, had undergone at least one ECG. After exclusion and restriction to normal sinus rhythm, 1300 (36.3%) cases (2264 ECGs) and 5200 (4.5%) controls (7922 ECGs) were retained. Because sex does not vary within an individual, it was balanced almost exactly (standardized mean difference [SMD] 0.02 in the development cohort and 0.01 in the external cohort). Age, in contrast, varies across a given patient’s ECGs, and matching on the median therefore left a residual imbalance at the ECG level, which is the unit of analysis (SMD 0.50 and 0.58, respectively). Because chronological age is the dominant predictor in this setting, we did not rely on the propensity-score matched set alone and additionally constructed an exact-age 1:1–matched case-control set (see *Temporal Label Trajectory Analysis* sections). The index date was the date of initial diagnosis for the cases; for the controls, the ECG acquisition date corresponded to their matched median age. The index date for cases was defined as the date of the first qualifying diagnostic code within the study period; when a patient had multiple qualifying codes, only the earliest was used. Because the controls had no observed diagnosis date and their index date was constructed rather than observed, the analysis was framed as case-control discrimination rather than time-to-event (incidence) prediction, and the preindex time windows were interpreted accordingly. In the matched Severance cohort, a patient-level split was used for training and internal validation, whereas the YSH cohort was reserved for external validation.

The primary analysis evaluated whether ECGs recorded before diagnosis could identify patients who would subsequently develop cognitive impairment or dementia within 10 years (cases) compared with controls. All case ECGs were acquired during the prediagnostic period, within the 10 years preceding the initial diagnosis. The temporal evolution of the ECG features across the prediagnostic period was examined separately (temporal label trajectory analysis).

### Data Preprocessing

For cohort characterization, each tabular EMR variable was summarized using the measurement closest to the index date within a 365-day period. The ECG waveforms were harmonized as follows: all recordings were high-pass-filtered to remove baseline wander, low-pass-filtered to suppress high-frequency noise, notch-filtered at 60 Hz to remove power line interference, resampled to 100 Hz, restricted to 12 leads, and *z*-normalized. Waveform representations were derived from the KED model using an ECG encoder, yielding an embedding sequence for each ECG for downstream classification [19]. The KED model additionally outputs prediction probabilities for 105 ECG interpretation labels, which were used for the temporal label trajectory analysis.

The KED encoder is a 1D residual convolutional network (xresnet1d101) that maps the preprocessed 12-lead waveform to a 768D embedding summarizing the entire recording. This embedding, rather than any hand-engineered measurement, such as QT interval or QRS duration, is the representation carried forward, and it is used in three ways. First, the encoder is fine-tuned end to end with a sigmoid classification head to produce the ECG-only probability of cognitive impairment and dementia. Second, the embedding is passed to the KED interpretation heads, which output probabilities for 105 ECG interpretation labels; these probabilities provide the clinically nameable view of the representation and are used in the temporal label-trajectory analysis. Third, the embedding is decomposed with respect to chronological age (see the *Age Decomposition and Exact-Age-Matched Evaluation* section) so that its age-independent component can be evaluated on its own. Because the representation is learned from the whole waveform rather than from predefined intervals, it can encode morphological variation that a conventional reading would summarize as normal sinus rhythm.

### Model Development

All models were developed on the Severance cohort and randomly partitioned at the patient level into training and validation sets (8:2) to prevent leakage; the YSH cohort was held out entirely and served as the external validation (test) set. The ECG-only model fine-tuned an xresnet1d101 backbone initialized from KED-pretrained weights, taking the 12-lead waveform as input and producing a single sigmoid probability of cognitive impairment and dementia through a classification head consisting of dropout (rate 0.3) and a fully connected layer. The model was trained with a class-weighted binary cross-entropy loss, in which the positive class was weighted by the ratio of negative to positive samples in the training set. Optimization used the AdamW optimizer (weight decay 1 × 10^–4^) with a batch size of 128 and a maximum of 50 epochs. The learning rate of the classification head was linearly warmed up to 1 × 10^–4^ over the first 2 epochs, during which the backbone was frozen; the backbone was then unfrozen with a learning rate of 1 × 10^–5^, and both learning rates subsequently followed a cosine decay schedule. Early stopping was applied after 10 epochs without improvement in the validation area under the receiver operating characteristic curve (AUROC), and the checkpoint with the highest validation AUROC was retained. No hyperparameter tuning was performed using the external cohort. To benchmark against the dominant available risk factors, an age-only model used logistic regression with chronological age (in years) as the sole feature. To test whether the ECG added information beyond age, a probability-level (late) fusion model combined the ECG-only sigmoid probability and age as two features in the logistic regression. All logistic regression models were fitted to the training set and applied unchanged to the internal validation and external cohorts.

Several safeguards were applied against information leakage, in addition to the patient-level split described before. The classification threshold and all other operating decisions were fixed on the Severance validation set; the YSH cohort was evaluated once, after the models and the operating point had been finalized, and was not used for model selection at any stage. No patient contributed data to both cohorts; the two hospitals share a common patient identifier system, which allowed this to be verified directly. The public datasets used to pretrain the KED model do not overlap with either study cohort.

### Model Evaluation

All models were evaluated on the held-out Severance validation set (internal validation) and on the entire YSH cohort (external validation). Each ECG contributed one prediction, so patients with more than one qualifying ECG contributed multiple observations; CIs were therefore obtained using cluster bootstrap resampling at the patient level, as described later. Discrimination was quantified using AUROC and the area under the precision-recall curve (AUPRC). Threshold-dependent metrics (accuracy, sensitivity, specificity, and *F*_1_-score) were reported at a fixed operating point determined on the Severance validation set and applied unchanged to the YSH cohort. Calibration was assessed using Brier scores. Clinical utility was assessed using decision curve analysis (DCA), in which the net benefit was plotted across a clinically plausible range of threshold probabilities. To express the incremental contribution of the ECG-derived probability in operational terms, each model was additionally evaluated at fixed sensitivities from 70% to 95%. Thresholds were selected within each cohort to reach the target sensitivity, so the number of detected cases was identical across models and the entire difference appeared as a change in the false-positive rate. Absolute reductions in the false-positive rate (in percentage points, with 95% CIs from 2000 patient-level bootstrap replicates) and positive and negative likelihood ratios are reported in Multimedia Appendix 1. Between-model comparisons used DeLong’s test for paired AUROC differences and the continuous net reclassification improvement (NRI) to evaluate the incremental value of the fusion models over the age-only baseline. For all metrics, 95% CIs were constructed using patient-level bootstrap resampling with 1000 iterations for each evaluation set.

### Age Decomposition and Exact-Age-Matched Evaluation

To provide direct evidence for our first hypothesis that the embedding carries information not explained by chronological age, we decomposed the 768D embedding with respect to age. Using the training set, a regression predicting each embedding dimension from chronological age was fitted, and the age-residualized embedding was defined as the observed embedding minus its age-predicted value (residual = E − Ê). A standardized logistic regression classifier was trained on the residuals to predict cognitive impairment and dementia. Age regression and the classifier were fitted to the training set only and applied unchanged to the internal validation set and the external cohort to prevent leakage. The discrimination of the residualized embedding was quantified with AUROC; the significance of AUROC>0.5 was assessed using a one-sided Mann-Whitney *U* test on the predicted scores (equivalent to AUROC), and 95% CIs were obtained using patient-level bootstrap (1000 iterations, as before). As an interpretable complement, ECG-predicted age was estimated using ridge regression from the embedding (trained on controls), and its agreement with chronological age was summarized using Pearson and Spearman correlation coefficients and the mean absolute error (MAE). As a second, design-based approach to the same question, all three models were additionally evaluated in the exact-age 1:1–matched case-control set described before, in which age alone was uninformative by construction. No model was refitted, and the operating thresholds were unchanged; only the evaluation set differed.

### Temporal Label Trajectory Analysis

To characterize the temporal evolution of ECG features, while removing residual age confounding, this analysis was performed on an exact-age 1:1–matched case-control set constructed, in addition to the primary 1:4 propensity score matching: cases and controls were matched on the exact age within the institution. Age balance was confirmed both overall (case 66.50 vs control 66.49 years; standardized mean difference [SMD]≈0) and within each prediagnostic window (|SMD|<0.12 in all four windows) so that the age balance underlying this design was maintained throughout the trajectory rather than only on average. In this age-matched set, we analyzed the prediction probability trajectories of the KED interpretation labels over the prediagnostic period, with all label probabilities being natural log-transformed.

Features were selected according to whether their case-control differences widened as the diagnosis approached. As a patient could contribute to multiple ECGs, the unit of observation was a single ECG, and all models used cluster-robust SEs clustered on the patient to account for the within-patient correlation. For each label, an ordinary least-squares model was used to regress the log probability on the case-control group, continuous time before diagnosis, and their interaction; the group-by-time interaction tested whether a case-control difference changed over time. To quantify the overall magnitude of this change, the case-control effect size at the two ends of the prediagnostic period—the earliest available year (9-10 years before diagnosis) and the final year (0-1 year before diagnosis)—was compared, and the net widening was summarized as Δd = d(0-1 year) − d(9-10 years), the difference in case-control Cohen d (on log probabilities) between these two endpoints. A difference was used rather than a ratio, which became unstable when the earlier effect size approached zero. A label qualified if its interaction was significant after Benjamini-Hochberg correction across the 105 labels (q<.05), and its Δd was at least 0.15.

To localize when each qualifying feature diverged, the prediagnostic period was partitioned into four windows: remote (5-10 years), intermediate (3-5 years), proximal (1-3 years), and imminent (0-1 year) before the index date. Within each window, the case-control effect size (Cohen d on log-transformed probabilities) was computed, and a two-sided Mann-Whitney *U* test, with Benjamini-Hochberg correction was applied across labels (q<.05). As these tests were applied to previously selected labels, they are reported as a descriptive localization rather than independent confirmation. To visualize these changes at the waveform level, a variational autoencoder trained on the same ECG waveforms was used to reconstruct a mean waveform for each group and prediagnostic window. The details are described in Multimedia Appendix 2.

As a sensitivity analysis, model discrimination was also evaluated within each prediagnostic window in the external cohort, comparing cases and controls whose ECGs were recorded in the same window.

### Statistical Analysis

Group differences in continuous variables were assessed with the Mann-Whitney *U* test, with effect sizes expressed as Cohen d. All hypothesis tests were two sided with α=.05, and multiplicity was controlled with the Benjamini-Hochberg false discovery rate, where indicated. Reporting followed the TRIPOD+AI (Transparent Reporting of a Multivariable Prediction Model for Individual Prognosis or Diagnosis + Artificial Intelligence) guidelines, with the completed checklist provided in Multimedia Appendix 3. Analyses were conducted in Python 3.10 using PyTorch, scikit-learn, statsmodels, and SciPy.

### Ethical Considerations

This study was approved by the Institutional Review Board of Severance Hospital (IRB #9-2023-0075). The requirement for informed consent was waived because of the retrospective nature of the study and the use of anonymized data.

## Results

### Baseline Characteristics

The flow of participants through screening and sequential exclusions at both sites is shown in Figure 1. Table 1 summarizes the matched development (Severance) and external (YSH) cohorts. The Severance cohort comprised 10,471 (40.9%) cases (patients who developed cognitive impairment or dementia) with 26,837 ECGs and 41,884 (3.8%) controls with 58,764 ECG records, and the YSH cohort comprised 1300 (36.3%) cases with 2264 ECG records and 5200 (4.5%) controls with 7922 ECG records. At both sites, patients who later developed dementia were older than those who did not (Severance: median 69, IQR 60-75, vs median 65, IQR 56-72 years; YSH: median 72, IQR 64-78, vs median 69, IQR 60-79 years); this residual age difference despite matching motivated the age-residualized analyses that followed. The detailed characteristics are presented in Table 1.

**Figure 1:**
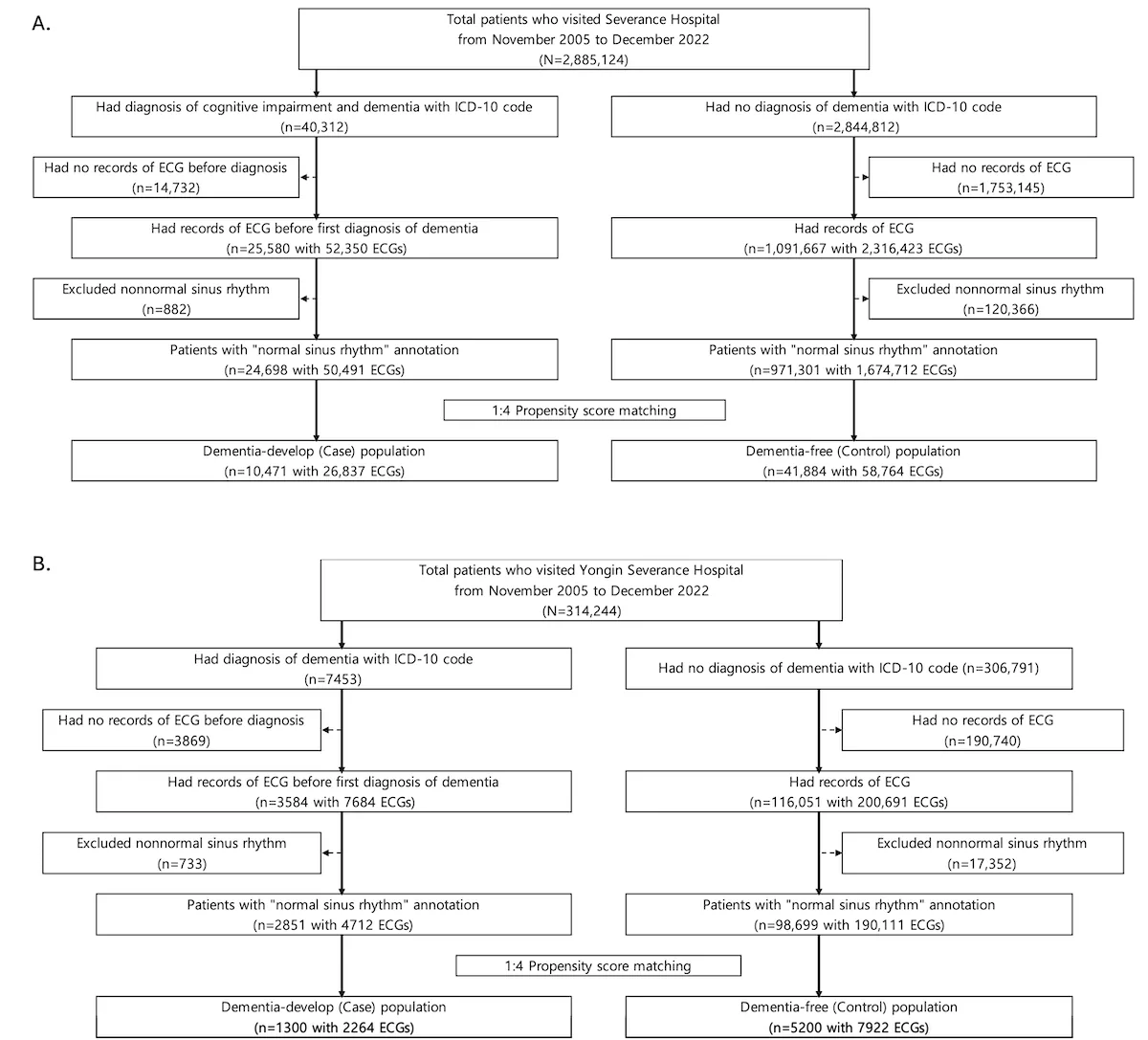
Flow diagram of participant selection. (A) Severance Hospital (development and internal validation cohorts) and (B) YSH (external validation cohort). Patients with at least one ECG between November 2005 and December 2022 were screened, and sequential exclusions were applied to retain only normal sinus rhythm ECGs. ECG: electrocardiogram; ICD-10: *International Statistical Classification of Diseases and Related Health Problems, Tenth Revision*; YSH: Yongin Severance Hospital.

**Table 1:** Baseline characteristics of study cohorts.

Characteristics			Severance			YSH^a^	
		Case (n=26,837 ECGs^b^)		Control (n=58,764 ECGs)	Case (n=2264 ECGs)		Control (n=7922 ECGs)
**Demographics**							
	Age (years), median (IQR)	69 (60-75)		65 (56-72)	72 (64-78)		69 (60-79)
	Sex (female), n (%)	15,387 (57.3)		34,292 (58.4)	1331 (58.8)		4680 (59.1)
**Alcohol status, n (%)**							
	Current	3514 (13.1)		7614 (13.0)	324 (14.3)		1103 (13.9)
	Ex	4976 (18.5)		10,093 (17.2)	225 (9.9)		915 (11.6)
	Never	15,206 (56.7)		31,239 (53.2)	1224 (54.1)		4373 (55.2)
**Smoking status, n (%)**							
	Current	1550 (5.8)		3858 (6.6)	394 (17.4)		1188 (15.0)
	Ex	3788 (14.1)		11,801 (20.1)	250 (11.0)		871 (11.0)
	Never	14,165 (52.8)		33,262 (56.6)	1288 (56.9)		3739 (47.2)
**Past history, n (%)**							
	Diabetes mellitus	7901 (29.4)		15,919 (27.1)	416 (18.4)		1870 (23.6)
	Heart disease	7689 (28.7)		14,873 (25.3)	341 (15.1)		1595 (20.1)
	Hepatitis	2925 (10.9)		7304 (12.4)	156 (6.9)		828 (10.5)
	Hypertension	8341 (31.1)		20,532 (34.9)	424 (18.7)		2605 (32.9)
	Hyperlipidemia	805 (3.0)		2217 (3.8)	40 (1.8)		216 (2.7)
	Stroke	1913 (7.1)		6064 (10.3)	90 (4.0)		689 (8.7)

^a^YSH: Yongin Severance Hospital.

^b^ECG: electrocardiogram.

### Discriminating Future Cognitive Impairment and Dementia Cases From Matched Controls Using Prediagnostic ECGs

All ECGs were recorded within 10 years of the initial diagnosis. Using these prediagnostic ECGs, chronological age alone discriminated cases from controls in both cohorts (age-only logistic regression: internal AUROC 0.811, 95% CI 0.804-0.818; external AUROC 0.809, 95% CI 0.799-0.819). When applied to the same prediagnostic ECGs, the fine-tuned ECG-only model achieved an internal AUROC of 0.803 (95% CI 0.797-0.810) and an external AUROC of 0.774 (95% CI 0.763-0.784), with AUPRCs of 0.666 and 0.514, respectively. Thus, discrimination was comparable to but did not exceed that of chronological age alone. Combining the two, the probability-level (late) fusion of the ECG-derived probability with age achieved the highest discrimination among the three models (internal AUROC 0.825, 95% CI 0.819-0.831; external AUROC 0.825, 95% CI 0.816-0.834). The performance metrics for all three models in both cohorts are presented in Table 2. Relative to the age-only baseline, this gain was statistically significant (DeLong ΔAUROC +0.014 internal and +0.016 external; both *P*<.001), with positive continuous NRI (+0.54, 95% CI 0.51-0.57 internal; +0.58, 95% CI 0.54-0.63 external). Calibration and clinical utility differed between the cohorts. Internally, all three models were well calibrated, tracking the diagonal across the predicted probability range, and the fusion model had the lowest Brier score (0.152; range 0.152-0.173 across models and cohorts; Figure 2A). Externally, discrimination was preserved (fusion AUROC 0.825), but calibration degraded; predicted probabilities systematically exceeded observed event fractions across the range (Figure 2C), indicating overprediction consistent with a between-site distribution shift and remediable by site-specific recalibration. In DCA, the fusion model provided the greatest net benefit internally across threshold probabilities of approximately 5%-50% (Figure 2B). Externally, all models yielded a positive net benefit over the treat-all and treat-none strategies across the lower threshold range relevant to screening and triage (approximately 5%-25%), while the fusion model retained benefits to slightly higher thresholds than age or ECG alone; above approximately 30%, the net benefit was limited, and the ECG-only curve fell below the treat-none strategy (Figure 2D). At a fixed sensitivity of 95%, adding the ECG-derived probability to age reduced the false-positive rate from 75.2% to 62.7% internally (absolute reduction 12.6 percentage points, 95% CI 9.7-15.6) and from 60.6% to 57.5% externally (absolute reduction 3.1 percentage points, 95% CI −1.3 to 5.5). At sensitivities of 90% and 85%, the reduction remained smaller, but its CI excluded zero in both cohorts (internal 4.3 and 3.0 percentage points, respectively; external 2.1 and 3.1 percentage points, respectively), whereas at sensitivities of 80% or lower, the reductions were below 1.5 percentage points, with CIs including zero. Positive likelihood ratios ranged from 1.26 to 3.51 and were below 2 at the highest sensitivities (Tables S1 and S2 in Multimedia Appendix 1). A sensitivity analysis in the external cohort supported these findings: excluding the 35 (0.7%) of 5200 control patients with evidence of unrecorded dementia did not materially change performance (fusion AUROC 0.829 vs 0.825) or the incremental discrimination over age (+0.016 vs +0.016), and these controls had already received case-like predicted probabilities (median 0.85 vs 0.38 for other controls; Tables S1 and S2 in Multimedia Appendix 4).

**Table 2:** Discriminative performance of ECG^a^- and age-based feature combinations for prediagnostic cognitive impairment and dementia in the internal and external validation cohorts^b^.

Dataset and features		AUROC^c^	AUPRC^d^	Accuracy	Sensitivity	Specificity	PPV^e^	NPV^f^	*F*_1_-score	Brier score
**Internal validation (Severance)**										
	ECG only	0.8033 (0.7968-0.8099)	0.6664 (0.6540-0.6692)	0.7406 (0.7344-0.7469)	0.7408 (0.7296-0.7530)	0.7405 (0.7331-0.7490)	0.5651 (0.5545-0.5761)	0.8625 (0.8560-0.8687)	0.6411 (0.6314-0.6505)	0.1601 (0.1572-0.1629)
	Age only	0.8108 (0.8038-0.8182)	0.6624 (0.6481-0.6764)	0.7645 (0.7578-0.7713)^g^	0.7180 (0.7057-0.7299)	0.7857 (0.7780-0.7935)^g^	0.6040 (0.5923-0.6161)^g^	0.8596 (0.8530-0.8663)	0.6561 (0.6455-0.6660)	0.1561 (0.1530-0.1591)
	ECG probability+age	0.8249 (0.8186-0.8313)^g^	0.6780 (0.6639-0.6916)^g^	0.7588 (0.7523-0.7654)	0.7456 (0.7339-0.7581)^g^	0.7647 (0.7574-0.7724)	0.5906 (0.5796-0.6018)	0.8685 (0.8619-0.8755)^g^	0.6591 (0.6499-0.6694)^g^	0.1519 (0.1490-0.1548)^g^
**External validation (YSH^h^)**										
	ECG only	0.7735 (0.7630-0.7839)	0.5137 (0.4913-0.5344)	0.6186 (0.6092-0.6281)	0.8187 (0.8019-0.8347)	0.5613 (0.5499-0.5723)	0.3483 (0.3350-0.3613)	0.9154 (0.9077-0.9238)	0.4887 (0.4745-0.5023)	0.1730 (0.1692-0.1764)
	Age only	0.8093 (0.7994-0.8193)	0.5153 (0.4923-0.5380)	0.7210 (0.7121-0.7301)^g^	0.7538 (0.7359-0.7710)	0.7117 (0.7014-0.7218)^g^	0.4281 (0.4114-0.4444)^g^	0.9099 (0.9025-0.9169)	0.5460 (0.5295-0.5614)	0.1515 (0.1479-0.1551)^g^
	ECG probability+age	0.8253 (0.8158-0.8342)^g^	0.5576 (0.5341-0.5811)^g^	0.7013 (0.6927-0.7103)	0.8329 (0.8170-0.8474) ^g^	0.6636 (0.6534-0.6746)	0.4148 (0.3991-0.4295)	0.9328 (0.9263-0.9391)^g^	0.5538 (0.5379-0.5678)^g^	0.1520 (0.1482-0.1556)

^a^ECG, electrocardiogram.

^b^Data are presented as point estimates with 95% CIs in parentheses, calculated using bootstrap resampling with 1000 iterations.

^c^AUROC: area under the receiver operating characteristic curve.

^d^AUPRC: area under the precision-recall curve.

^e^PPV: positive predictive value.

^f^NPV: negative predictive value.

^g^Indicates the best performance for each metric.

^h^YSH: Yongin Severance Hospital.

**Figure 2:**
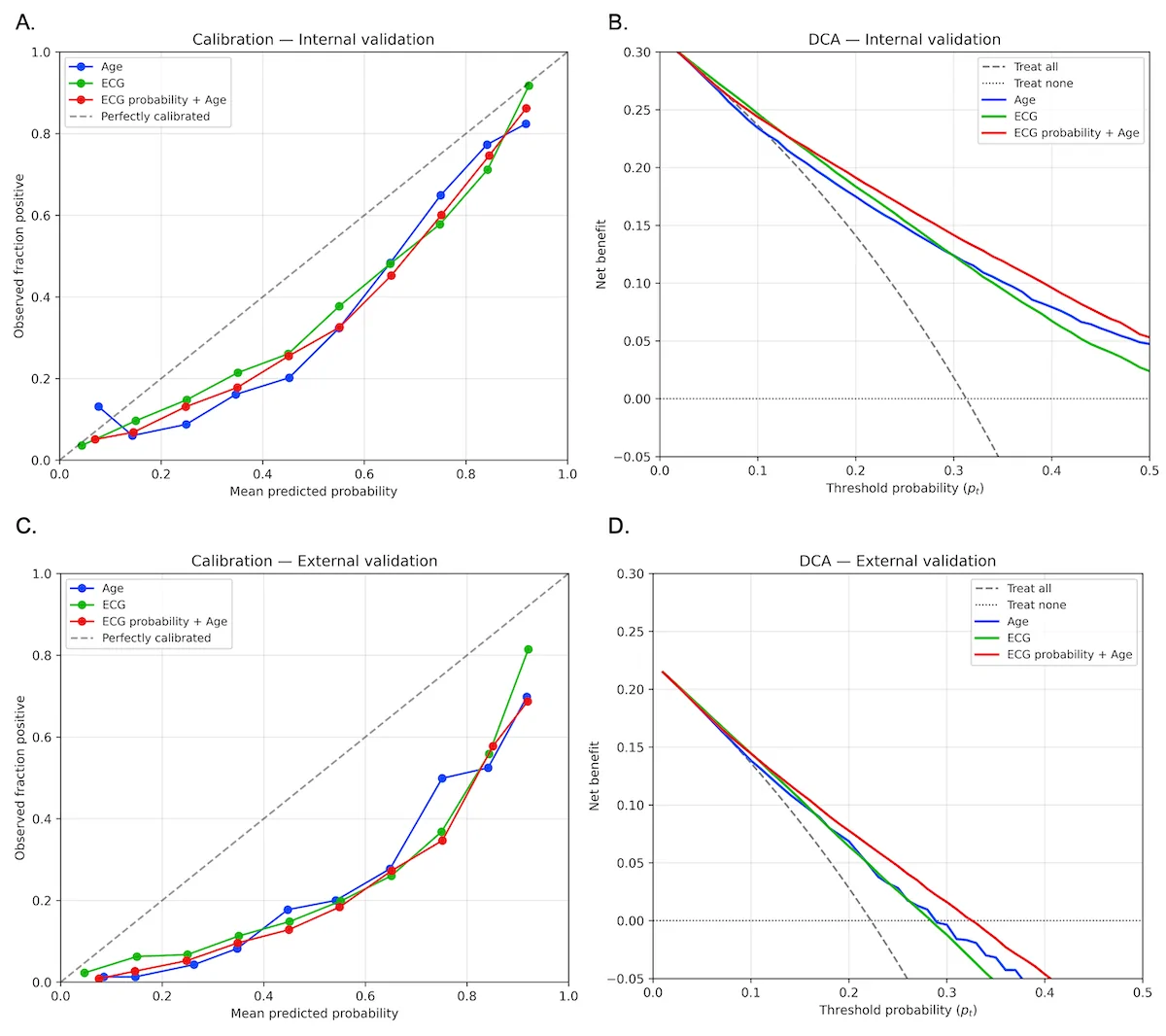
Calibration and DCA for cognitive impairment and dementia discrimination models. (A,C) Calibration plots and (B,D) DCA for age-only, ECG-only, and ECG probability+age models in (top, A,B) internal validation (Severance) and (bottom, C,D) external validation (YSH) cohorts. In the calibration plots, the diagonal dashed line indicates perfect calibration (predicted=observed); points closer to this line indicate better calibration. In the decision curve plots, the dashed and dotted reference curves represent treat-all and treat-none strategies, respectively, and a model is considered useful in the threshold range over which its net benefit curve lies above both reference strategies. DCA: decision curve analysis; ECG, electrocardiogram; YSH, Yongin Severance Hospital.

### Age Decomposition and Exact-Age Matching: ECG Information Beyond Chronological Age

ECG-predicted age (a regression model mapping the 768D embedding to chronological age, fitted in controls) correlated with chronological age in both cohorts (internal: Pearson r=0.662, MAE 7.3 years; external: Pearson r=0.638, MAE 7.7 years). Each embedding dimension was then regressed on chronological age, and age-residualized embedding (residual after removing the age-predictable component) was used to classify cognitive impairment and dementia. The age-residualized embedding yielded an internal AUROC of 0.590 (95% CI 0.581-0.599) and an external AUROC of 0.579 (95% CI 0.567-0.592), both were >0.5 (one-sided Mann-Whitney *U* test, *P*<.001; 95% CIs from bootstrap resampling). In the exact-age 1:1–matched set, age alone was uninformative by construction (internal: AUROC 0.501, 95% CI 0.488-0.514; external: AUROC 0.500, 95% CI 0.482-0.518). The ECG-only model, nevertheless, discriminated cases from controls above chance in both cohorts (internal: AUROC 0.632, 95% CI 0.619-0.646; external: AUROC 0.578, 95% CI 0.558-0.597), whereas the fusion model performed less well (internal: AUROC 0.554, 95% CI 0.541-0.567; external: AUROC 0.539, 95% CI 0.521-0.558), as expected because the age component carries no information in this set by design (Table S1 in Multimedia Appendix 5).

### Temporal Label Trajectory Analysis

On the exact-age 1:1–matched set, the mean age did not differ between cases and controls, either overall (66.50 vs 66.49 years) or within any prediagnostic window. Of the 105 KED interpretation labels, 19 (18.1%) qualified, each showing a case-control difference that widened significantly toward diagnosis (patient-clustered group-by-time interaction, Benjamini-Hochberg q<.05) and met the prespecified effect-size threshold. Figure 3 presents these 19 labels as a heatmap of the standardized case-control difference (Cohen d) across the four prediagnostic windows; the color gradient shows the divergence increasing progressively from the remote to the imminent window. Consistent with this pattern, almost all qualifying labels showed the largest separation in the imminent (0-1-year) window, with significance concentrated in the proximal (1-3-year) and imminent windows. The qualifying labels were dominated by nonspecific ST segment and T wave changes (lateral, inferior, and septal distributions), acute ischemic labels (acute myocardial infarction and acute ST-segment-elevation myocardial infarction), atrial and junctional arrhythmias (atrial flutter, ectopic atrial rhythm, and accelerated junctional rhythm), and left and right atrial abnormalities. To examine these label-level changes at the waveform level, mean waveforms decoded from the variational autoencoder representation were plotted by prediagnostic window in leads I, II, V1, and V5. The window-to-window differences were confined to the ST segment and T wave, with little change in QRS amplitude or duration, and were most evident in the lateral and inferior leads (I, II, and V5); smaller differences were also visible in the P wave segment in leads II and V1. This distribution parallels the label-level findings in Figure 3, in which nonspecific lateral and inferior ST segment and T wave labels showed the largest divergence, followed by atrial and junctional labels (Figure S1 in Multimedia Appendix 2). The per label effect sizes and window-specific statistics are provided in Table S1 in Multimedia Appendix 6, and individual label trajectories are shown in Figure 4.

**Figure 3:**
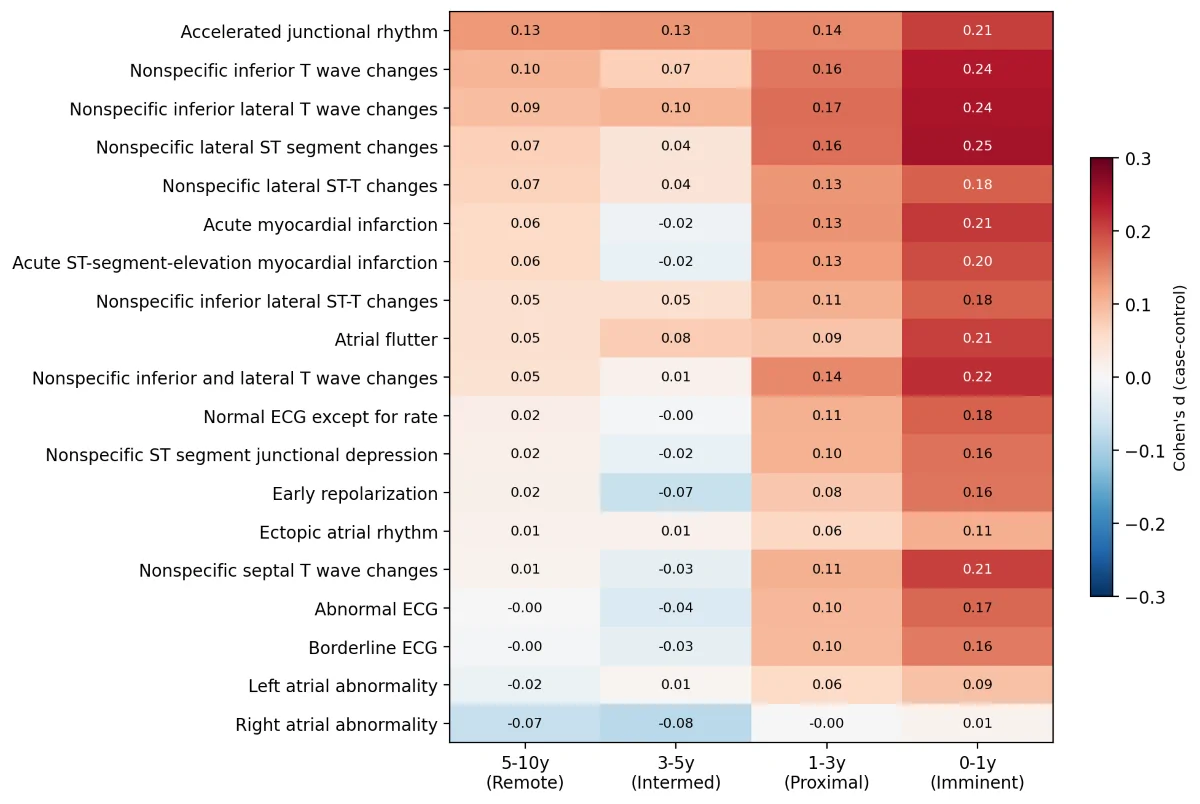
Temporal divergence of significant ECG features between case and control groups across four prediagnostic windows. Heatmap of 19 KED interpretation labels whose case-control difference widened significantly toward diagnosis (see the Methods section for selection criteria), on the exact-age (1:1)–matched set. Each row represents a qualifying label, and each column represents a prediagnostic window: remote (5-10 years), intermediate (3-5 years), proximal (1-3 years), and imminent (0-1 year) before the index date. Color encodes Cohen d on log-transformed KED prediction probabilities (red, higher in cases; blue, higher in controls), and the value of Cohen d is shown in each cell. Labels are ordered by the remote window effect size, and the gradient shows divergence increasing from the remote to the imminent window. ECG, electrocardiogram; KED, knowledge-enhanced diagnosis.

**Figure 4:**
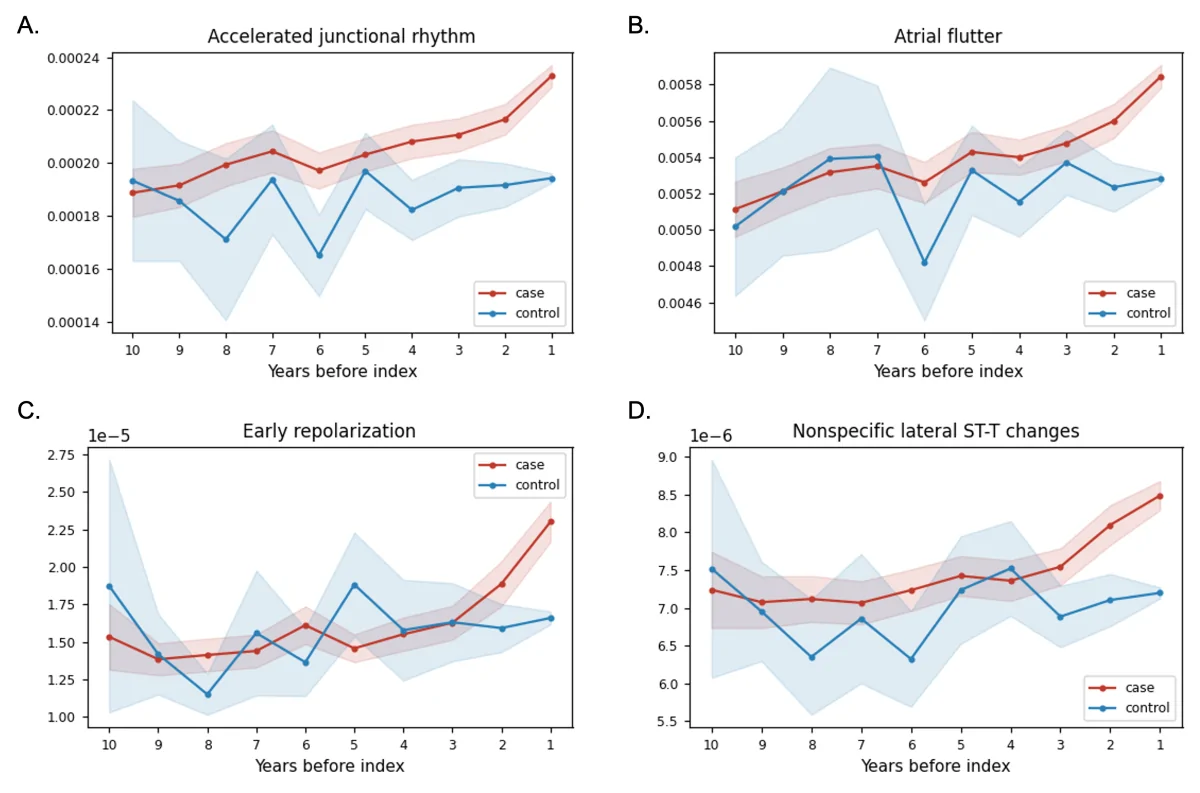
Temporal trajectories of KED-predicted probabilities for four representative ECG features in case and control groups. Red and blue lines indicate the case and control groups, respectively, and shaded bands represent 95% CIs. (A) Accelerated junctional rhythm, (B) atrial flutter, (C) early repolarization, and (D) nonspecific lateral ST-T changes. The x-axis represents years before the index date, and the right side corresponds to the period imminent to diagnosis. ECG: electrocardiogram; KED: knowledge-enhanced diagnosis.

Because ECGs were unevenly distributed across windows, we also examined whether the overall model performance was driven by ECGs recorded shortly before diagnosis. In a sensitivity analysis of the external validation cohort, in which cases and controls were compared within the same prediagnostic window, the fusion model showed similar discrimination across windows (AUROC 0.822, 95% CI 0.802-0.840, to 0.844, 95% CI 0.818-0.869) and exceeded age alone in every window (Table S1 in Multimedia Appendix 7).

## Discussion

### Principal Findings

In this two-site retrospective study, foundation model embeddings of routine ECGs carried information about future cognitive impairment and dementia cases that was largely shared but not fully explained by chronological age. Chronological age alone strongly discriminated cases from matched controls (external AUROC 0.809), and the ECG-only model did not exceed it (external AUROC 0.774). Nonetheless, adding the ECG-derived probability to age improved discrimination over age alone, with a statistically significant gain (external AUROC 0.825 vs 0.809; DeLong *P*<.001), accompanied by a positive continuous NRI. The model’s performance was maintained in an external cohort from a different clinical setting. Severance is a tertiary referral center in central Seoul, whereas the YSH is a general hospital serving a suburban population, and the two cohorts differed in age (Table 1). External validation was therefore performed in a distinct patient population rather than in a resplit of the development data, supporting the transportability of the model across care settings within the Korean health care system. The central positive finding was that age-residualized embedding, obtained by removing the age-predictable component from each embedding dimension, remained predictive of cognitive impairment and dementia in both internal and external validations (AUROC 0.590 and 0.579; both *P*<.001). Therefore, the embedding encodes aging-related electrophysiological information that is complementary to and not fully explained by chronological age. Fusion with age also showed acceptable calibration in the internal cohort and a positive net benefit across clinically relevant threshold probabilities. Together, these results suggest that routine ECG is not a stand-alone diagnostic test or an age-independent electrical signature of dementia but a low-cost, age-complementary signal that could triage individuals toward confirmatory cognitive or biomarker assessment. Complementing these predictive results, a label trajectory analysis showed that case-control divergence was not static but accelerated toward diagnosis. Additionally, 19 ECG interpretation labels, spanning nonspecific ST segment and T wave (repolarization) changes, atrial and junctional patterns, and infarct-like patterns, widened significantly and reached their greatest separation in the proximal (1-3 years) and imminent (0-1 year) windows.

### Comparison With Prior Work

The fact that ECGs read as clinically normal still carried predictive information indicates that subclinical electrophysiological tendencies accompany the prediagnostic period of dementia. Previous studies have linked individual ECG features, such as QT prolongation, ST-T abnormalities, and reduced heart rate variability, to cognitive decline [13,18]. Our learned representation integrates information across the entire waveform and, through age residualization, suggests that its dementia-relevant component is not entirely explained by chronological age. Rather than maximizing discrimination or estimating ECG age [21,22], we showed that routine ECG embeddings carry information complementary to chronological age and characterized how related ECG patterns evolve as diagnosis approaches. Because all included ECGs were clinically interpreted as normal sinus rhythm and ECGs flagged as abnormal were excluded, the KED label probabilities represent a subclinical shift toward the named patterns rather than their overt presence. Therefore, we described these as tendencies rather than attributing specific cardiac diseases to our participants.

Within this constraint, qualifying labels were grouped into three families whose clinical counterparts were linked to cognition. The dominant and earliest-emerging shifts were toward nonspecific ST segment and T wave patterns. Ventricular repolarization is sensitive to cardiac autonomic tone, and lower heart rate variability has been associated with cognitive decline [23], making autonomic involvement a possible explanation, although autonomic function was not measured directly in this study. The second family comprised atrial and junctional patterns, including atrial flutter, ectopic atrial and accelerated junctional rhythms, and left atrial abnormalities. These tendencies are compatible with the electrophysiological changes described in atrial cardiopathy, a remodeled atrial substrate linked to cognitive impairment, even in the absence of atrial fibrillation [24], and abnormal P wave indices predict dementia independent of atrial fibrillation [25]. The third family comprised infarct-like patterns, including acute myocardial infarction and ST segment elevation labels. On ECGs read as clinically normal, these should be interpreted only as a subtle resemblance to the model-recognized infarct-like morphology rather than evidence of myocardial infarction or ischemia. Therefore, the clinical implications of these findings are unclear. Prior studies have linked subclinical myocardial injury, measured with high-sensitivity troponin, with cerebral small-vessel disease and vascular cognitive decline [26]; however, whether the model-derived patterns observed here reflect a related process cannot be determined from our data.

These families intensified as the diagnosis date approached, rather than showing uniform separation during the preceding decade. This pattern is compatible with the broader heart-brain axis literature and suggests that subtle cardiac electrophysiological changes may accompany the prediagnostic phase of dementia [27]. We cannot determine whether late intensification reflects evolving cardiac or systemic changes, processes associated with emerging cognitive disease, or differences in clinical testing and ascertainment as patients approach diagnosis. However, in the age-matched cohort used for the trajectory analysis, the number of ECGs per patient was 1.4-1.6 times higher in cases than in controls in every prediagnostic window, not just in the window immediately preceding diagnosis (Table S2 in Multimedia Appendix 6), suggesting a general difference in health care use rather than an intensification of testing confined to the period around diagnosis. In addition, in the external validation cohort, discrimination was similar across all four windows when cases and controls were compared within the same window, indicating that the overall model performance was not driven only by ECGs recorded shortly before diagnosis.

### Clinical Implementation Considerations

If these findings are translated into practice, the model could serve as a screening tool in routine care, rather than as a diagnostic tool. A risk score could be computed automatically whenever a 12-lead ECG is recorded for another clinical indication, without an additional cost or patient burden. Individuals exceeding a predefined threshold could then be referred for a brief cognitive screening test, such as the Mini-Mental State Examination or the Montreal Cognitive Assessment. This approach may help identify individuals who warrant further evaluation earlier, providing an opportunity for timely intervention. Before clinical use, however, site-specific recalibration would be required, as calibration was lower in the external cohort (Figure 2C), and prospective studies are needed to determine whether such a screening pathway improves patient outcomes.

### Limitations

This study had some limitations. First, cognitive impairment and dementia were ascertained solely from ICD-10 codes and were not independently adjudicated using detailed clinical records, standardized cognitive assessments, or biomarker or neuropathological confirmation; therefore, some degree of outcome misclassification, particularly of prodromal stages, may remain and may have introduced label noise. Second, the case-control differences in ECGs were concentrated in the year before diagnosis rather than being spread across the preceding decade. This raises the possibility that part of the signal arises around the time of diagnosis itself, for example, from early disease processes already affecting the heart or from more frequent testing and incidental findings as patients approach diagnosis, rather than representing a truly early antecedent marker. The age-residualized analysis does not rely on how close an ECG is to the diagnosis date; therefore, it reduces but does not fully remove this concern. A related concern is that the date on which a diagnosis code is first entered may lag the onset of symptoms by months, so the window labeled “imminent” may contain ECGs recorded after clinical suspicion had already prompted additional testing. Differences in care-seeking behavior and coding lag cannot be fully excluded. Third, both cohorts were drawn from a single academic health system in South Korea and were recorded on equipment from a single manufacturer; therefore, the findings require validation in multiethnic and multinational populations before broader generalization. In addition, both sites, Severance and YSH, provide secondary or tertiary care, in which the prevalence of cognitive impairment and dementia is likely to be higher than in the general population; the predictive values reported here therefore cannot be transferred directly to a community screening setting. Fourth, because only ECGs interpreted as normal sinus rhythm were included, the findings may not apply to patients with abnormal ECGs, such as those with atrial fibrillation or conduction abnormalities, in whom the risk of cognitive impairment and dementia may be higher.

### Conclusion

In the two cohorts, adding routine ECG foundation model embeddings to chronological age modestly but consistently improved the prediagnostic discrimination of future cognitive impairment and dementia. This gain reflected the aging-related electrophysiological information carried by the embeddings, which was complementary to and not fully explained by chronological age. This information was dynamic rather than static and intensified as the diagnosis approached, accompanied by shifts toward repolarization, atrial, and ischemic ECG patterns. Because the case-control separation was concentrated in the period closest to diagnosis rather than sustained across the preceding decade, these embeddings are better understood as a proximity-weighted triage signal than as a stable decade-long biomarker of dementia risk. We cannot determine whether this late intensification reflects evolving cardiac or systemic changes, processes associated with emerging cognitive disease, or differences in clinical testing and ascertainment as patients approach diagnosis. Together, these findings suggest that routine ECG is a low-cost, age-complementary triage signal for cognitive impairment and dementia screening, rather than a stand-alone diagnostic test. Prospective multiethnic validation, biomarker-confirmed outcomes, subtype-stratified analyses, and evaluation of community screening prevalence are needed before clinical deployment.

## Data Availability

Data were obtained under institutional data use agreements and cannot be shared publicly owing to privacy regulations. The analysis codes are available from the corresponding author upon reasonable request.

## Appendix

Multimedia Appendix 1 Clinical interpretation of the incremental gain over age at fixed sensitivity.

Multimedia Appendix 2 Variational autoencoder-based analysis of ECG morphology. ECG: electrocardiogram.

Multimedia Appendix 3 TRIPOD+AI (Transparent Reporting of a Multivariable Prediction Model for Individual Prognosis or Diagnosis + Artificial Intelligence) reporting checklist.

Multimedia Appendix 4 Sensitivity analysis for potential misclassification of control patients (external validation cohort).

Multimedia Appendix 5 Model performance in the exact-age 1:1–matched case-control set.

Multimedia Appendix 6 Prediagnostic trajectories of ECG interpretation labels in the exact-age-matched case-control set. ECG: electrocardiogram.

Multimedia Appendix 7 Assessment of temporal bias and proximity-to-diagnosis effects in the external validation cohort.

## Abbreviations

AUPRC: area under the precision-recall curve
AUROC: area under the receiver operating characteristic curve
DCA: decision curve analysis
ECG: electrocardiogram
EMR: electronic medical record
ICD-10: *International Statistical Classification of Diseases and Related Health Problems, Tenth Revision*
KED: knowledge-enhanced diagnosis
MAE: mean absolute error
NRI: net reclassification improvement
SMD: standardized mean difference
YSH: Yongin Severance Hospital

## Generative AI Use

During manuscript preparation, the authors used two generative artificial intelligence tools, ChatGPT (OpenAI) and Claude (Anthropic), for English language editing of author-written drafts, specifically to correct grammar and improve readability. The tools were not used at any stage of the research itself: They did not generate scientific content; formulate the study design; develop or run the analyses; interpret the results; or produce, complete, or verify any references or citations, all of which were compiled and checked by the authors. Every tool-assisted passage was subsequently reviewed and revised by the authors, who took full responsibility for the content of the published work.
