# Supplementary material for: Electrocardiogram Embeddings Beyond Chronological Age for Prediagnostic Cognitive Impairment and Dementia: Development and External Validation Study
Source: J Med Internet Res. 2026 Oct 23;28:e108392. doi: 10.2196/108392 (PMC13743964; doi:10.2196/108392)
Supplement: Supplementary file 1 [file jmir_v28i1e108392_app1.docx]

**Multimedia Appendix 1**

**Clinical interpretation of the incremental gain over age at fixed sensitivity**

Discrimination metrics such as the area under the receiver operating characteristic curve do not translate directly into clinical value. To express the incremental contribution of the ECG-derived probability in operational terms, each model was evaluated at a series of fixed sensitivities. Because sensitivity is held constant, the number of cases detected is identical across models by construction, and the entire difference appears as a change in the false-positive rate. The absolute reduction is reported in percentage points, following the convention used in comparable artificial intelligence screening studies.

**Table S1. Internal validation cohort (Severance)**

| **Sensitivity** | **FPR**  **Age only** | **FPR**  **ECG only** | **FPR**  **Fusion** | **Absolute**  **reduction** | **95% CI** | **LR+**  **Age → fusion** | **LR−**  **Age → fusion** |
| --- | --- | --- | --- | --- | --- | --- | --- |
| 95% | 75.2% | 68.1% | 62.7% | **12.6%*** | (+9.68, +15.64) | 1.26 → 1.52 | 0.202 → 0.134 |
| 90% | 51.6% | 57.7% | 47.3% | **4.3%*** | (+2.83, +6.34) | 1.74 → 1.90 | 0.207 → 0.190 |
| 85% | 40.0% | 48.7% | 37.0% | **3.0%*** | (+1.79, +5.88) | 2.12 → 2.30 | 0.250 → 0.238 |
| 80% | 31.1% | 37.7% | 30.1% | **1.1%** | (-0.09, +2.31) | 2.57 → 2.66 | 0.290 → 0.286 |
| 75% | 25.1% | 28.2% | 24.3% | **0.9%** | (-0.33, +2.11) | 2.99 → 3.09 | 0.334 → 0.330 |
| 70% | 20.1% | 22.7% | 20.0% | **0.1%** | (-0.69, +1.16) | 3.48 → 3.51 | 0.375 → 0.375 |

**Table S2. External validation cohort (Yongin Severance Hospital)**

| **Sensitivity** | **FPR**  **Age only** | **FPR**  **ECG only** | **FPR**  **Fusion** | **Absolute**  **reduction** | **95% CI** | **LR+**  **Age → fusion** | **LR−**  **Age → fusion** |
| --- | --- | --- | --- | --- | --- | --- | --- |
| 95% | 60.6% | 70.3% | 57.5% | **3.1%** | (-1.27, +5.47) | 1.57 → 1.65 | 0.127 → 0.118 |
| 90% | 46.9% | 57.7% | 44.8% | **2.1%*** | (+0.29, +6.10) | 1.92 → 2.01 | 0.188 → 0.181 |
| 85% | 39.0% | 48.9% | 35.9% | **3.1%*** | (+0.75, +5.95) | 2.18 → 2.37 | 0.246 → 0.234 |
| 80% | 31.2% | 41.0% | 30.0% | **1.2%** | (-0.41, +3.70) | 2.57 → 2.67 | 0.290 → 0.285 |
| 75% | 26.8% | 35.2% | 25.9% | **0.9%** | (-0.25, +3.40) | 2.80 → 2.90 | 0.341 → 0.337 |
| 70% | 22.5% | 30.4% | 22.0% | **0.5%** | (-0.19, +3.29) | 3.12 → 3.19 | 0.387 → 0.384 |

FPR = false-positive rate = 1 − specificity. The absolute reduction is the difference in false-positive rate between the fusion model and age alone, in percentage points; an asterisk marks comparisons whose 95% CI excludes zero. CIs are percentile intervals from 2,000 patient-level bootstrap replicates.

Thresholds were chosen within each cohort to reach the target sensitivity. Sensitivity is therefore fixed by design rather than estimated, and the CIs reflect variability in specificity only.

The ECG-only column is shown to make clear that the ECG-derived probability does not replace age: its false-positive rate exceeded that of age alone at every sensitivity in the external cohort and at all sensitivities except the highest in the internal cohort, where it was lower (68.1% vs 75.2% at 95% sensitivity). The gain is obtained only when the two are combined.

Positive likelihood ratios ranged from 1.26 to 3.51 and were below 2 at the highest sensitivities, so a positive result does not strongly confirm the outcome. The incremental value lies in reducing unnecessary evaluations at a fixed detection rate rather than in ruling the outcome in.

*Abbreviations:* CI, confidence interval; ECG, electrocardiogram; FPR, false-positive rate; LR+, positive likelihood ratio; LR−, negative likelihood ratio.
