# Supplementary material for: Electrocardiogram Embeddings Beyond Chronological Age for Prediagnostic Cognitive Impairment and Dementia: Development and External Validation Study
Source: J Med Internet Res. 2026 Oct 23;28:e108392. doi: 10.2196/108392 (PMC13743964; doi:10.2196/108392)
Supplement: Supplementary file 2 [file jmir_v28i1e108392_app2.docx]

Multimedia Appendix 2

Variational Autoencoder–Based Analysis of ECG Morphology

This appendix describes a complementary analysis in which a variational autoencoder (VAE) was used to visualize the ECG waveform characteristics that differed between cases and controls. The analysis was independent of the KED foundation model used in the main analyses.

# Methods

A VAE is an unsupervised deep learning model consisting of an encoder, which compresses the input ECG into a low-dimensional latent variable, and a decoder, which reconstructs the ECG from that variable. Unlike a standard autoencoder, the encoder outputs the mean and standard deviation of a Gaussian distribution for each latent dimension, and the model is trained to minimize both the reconstruction error and the Kullback–Leibler divergence from a standard Gaussian distribution [1]. This yields a continuous latent space in which each dimension (factor) can be varied to observe its effect on the reconstructed waveform.

The VAE was trained on ECG median-beat waveforms from Yongin Severance Hospital, excluding ECGs interpreted as "abnormal ECG" or "pacemaker" and all ECGs from patients included in the case or control groups of this study. The architecture was adopted from van de Leur et al [2]: the encoder, a one-dimensional convolutional neural network, takes 8 independent leads (I, II, V1–V6) as input and outputs a 32-dimensional latent variable, and the decoder, a one-dimensional transposed convolutional network, reconstructs the ECG from it.

The trained encoder was applied to the ECGs of cases and controls, and the latent means were used as 32 morphological factors per ECG. For each factor, the case–control difference was calculated as Cohen's d in each of the four pre-diagnostic windows. For representative factors, defined as those whose case–control difference increased toward the index date, median beats were reconstructed by the decoder with the factor set to the mean value of cases or controls in each window.

Because all included ECGs were interpreted as normal sinus rhythm, factor differences represent subtle morphological tendencies rather than overt abnormalities and should not be interpreted as evidence of specific cardiac disease.

**Figure S1. Reconstructed median beats for representative VAE factors by pre-diagnostic window.** For each factor, median beats were reconstructed with that factor set to the mean value of cases (top row, red) or controls (bottom row, blue) in each window and all other factors fixed at their overall mean. Darker shades indicate windows closer to the index date, from remote (5–10 years) to imminent (0–1 year). Leads I, II, V1, and V5 were selected a priori to represent the high lateral, inferior, septal, and lateral precordial territories, corresponding to the anatomical regions of the ECG phenotypes.

# References

1. Kingma DP, Welling M. Auto-encoding variational Bayes. arXiv:1312.6114. 2013.
2. van de Leur RR, Bos MN, Taha K, et al. Improving explainability of deep neural network-based electrocardiogram interpretation using variational auto-encoders. *Eur Heart J Digit Health*. 2022;3(3):390–404.
