# Supplementary material for: Electrocardiogram Embeddings Beyond Chronological Age for Prediagnostic Cognitive Impairment and Dementia: Development and External Validation Study
Source: J Med Internet Res. 2026 Oct 23;28:e108392. doi: 10.2196/108392 (PMC13743964; doi:10.2196/108392)
Supplement: Supplementary file 3 [file jmir_v28i1e108392_app3.docx]

**Multimedia Appendix 3**

**TRIPOD+AI reporting checklist**

| **Section / Topic** | **Item** | **Checklist item (TRIPOD+AI)** | **Reported in manuscript** |
| --- | --- | --- | --- |
| **TITLE** | | | |
| Title | **1** | Identify the study as developing or evaluating a multivariable prediction model, the target population, and the outcome to be predicted. | Title page. |
| **ABSTRACT** | | | |
| Abstract | **2** | Provide a structured abstract following the TRIPOD+AI for Abstracts checklist. | Abstract (Background, Objective, Methods, Results, Conclusions). |
| **INTRODUCTION** | | | |
| Background | **3a** | Explain the healthcare context (diagnostic vs prognostic) and rationale for developing/evaluating the model, with references to existing models. | Introduction, paragraphs 1–3. |
|  | **3b** | Describe the target population and intended purpose of the model in the care pathway, including intended users. | Introduction (final paragraph); Methods, Study Design. |
|  | **3c** | Describe any known health inequalities between sociodemographic groups. | Discussion, Limitations. |
| Objectives | **4** | Specify study objectives, including whether the study describes development, validation, or both. | Introduction (final paragraph). |
| **METHODS** | | | |
| Data | **5a** | Describe sources of data separately for development and evaluation datasets, the rationale, and representativeness. | Methods — Study Design and Data Sources. |
|  | **5b** | Specify the dates of participant data (accrual start/end; end of follow-up if applicable). | Methods — Study Design and Data Sources. |
| Participants | **6a** | Specify key elements of the study setting, including number and location of centres. | Methods — Study Design and Data Sources. |
|  | **6b** | Describe the eligibility criteria for study participants. | Methods — Participant Selection and Research Tasks. |
|  | **6c** | Give details of any treatments received and how they were handled, if relevant. | Not applicable.*.* |
| Data preparation | **7** | Describe data pre-processing and quality checking, including whether similar across sociodemographic groups. | Methods — Participant Selection / Model Development. |
| Outcome | **8a** | Define the outcome and time horizon, how/when assessed, rationale, and consistency across groups. | Methods — Participant Selection and Research Tasks. |
|  | **8b** | If outcome assessment requires subjective interpretation, describe assessor qualifications/characteristics. | Not applicable. |
|  | **8c** | Report any actions to blind assessment of the outcome. | Not applicable. |
| Predictors | **9a** | Describe the choice of initial predictors and any pre-selection before model building. | Methods — Model Development. |
|  | **9b** | Clearly define all predictors, including how and when measured (and any blinding). | Methods — Model Development |
|  | **9c** | If predictor measurement requires subjective interpretation, describe assessor qualifications. | Not applicable. |
| Sample size | **10** | Explain how study size was determined (separately for development/evaluation) and justify sufficiency. | Methods — Participant Selection and Research Tasks. |
| Missing data | **11** | Describe how missing data were handled; give reasons for omitting data. | Methods — Data Preprocessing. |
| Analytical methods | **12a** | Describe how data were used/partitioned in the analysis, considering sample-size requirements and leakage. | Methods — Model Development. |
|  | **12b** | Describe how predictors were handled (functional form, rescaling, transformation, standardization). | Methods — Model Development; Age Decomposition |
|  | **12c** | Specify model type, rationale, all model-building steps (incl. hyperparameter tuning) and internal validation. | Methods — Model Development, Age Decomposition, Statistical Analysis. |
|  | **12d** | Describe how heterogeneity across clusters was handled (see TRIPOD-Cluster). | Not applicable |
|  | **12e** | Specify all measures and plots (discrimination, calibration, clinical utility) and model-comparison methods. | Methods — Model Evaluation. |
|  | **12f** | Describe any model updating (e.g., recalibration) arising from evaluation. | Not applicable. |
|  | **12g** | For model evaluation, describe how predictions were calculated (formula, code, object, API). | Methods — Model Evaluation; Code availability. |
| Class imbalance | **13** | If class-imbalance methods were used, state why/how and any recalibration. | Methods — Model Development. |
| Fairness | **14** | Describe any approaches used to address model fairness and their rationale. | Discussion, Limitations. |
| Model output | **15** | Specify model output (probability, classification); detail and justify any thresholds. | Methods — Model Evaluation. |
| Development vs evaluation | **16** | Identify differences between development and evaluation data in setting, eligibility, outcome, predictors. | Methods — Study Design; Results — Table 1. |
| Ethical approval | **17** | Name the IRB/ethics committee and describe informed consent or its waiver. | Methods — Ethical Statement. |
| **OPEN SCIENCE** | | | |
| Funding | **18a** | Give the source of funding and role of funders. | Funding. |
| Conflicts of interest | **18b** | Declare conflicts of interest and financial disclosures for all authors. | Conflicts of Interest. |
| Protocol | **18c** | Indicate where the protocol can be accessed or state none was prepared. | Not reported. |
| Registration | **18d** | Provide registration information or state the study was not registered. | Not reported. |
| Data sharing | **18e** | Provide details of the availability of the study data. | Data Availability. |
| Code sharing | **18f** | Provide details of the availability of the analytical code. | Data Availability; Statistical Analysis. |
| **PATIENT & PUBLIC INVOLVEMENT** | | | |
| Patient & public involvement | **19** | Provide details of any patient/public involvement, or state none. | No patient or public involvement in design, conduct, or reporting. |
| **RESULTS** | | | |
| Participants | **20a** | Describe the flow of participants, numbers with/without the outcome, follow-up; a diagram may help. | Results — Figure 1 (participant flow diagram). |
|  | **20b** | Report characteristics overall and per data source (key dates, predictors, events, missingness). | Results — Table 1 (baseline characteristics, both sites). |
|  | **20c** | For evaluation, compare distributions of important predictors with the development data. | Results — Table 1 (development vs external comparison). |
| Model development | **21** | Specify the number of participants and outcome events in each analysis. | Results — Table 1 / task descriptions. |
| Model specification | **22** | Provide details of the full model to enable third-party prediction and evaluation. | Methods — Model Development; Code Availability. |
| Model performance | **23a** | Report performance estimates with confidence intervals, incl. key subgroups; use plots. | Results — Table 2 |
|  | **23b** | If examined, report heterogeneity in performance across clusters. | Not applicable. |
| Model updating | **24** | Report the results of any model updating. | Not applicable. |
| **DISCUSSION** | | | |
| Interpretation | **25** | Give an overall interpretation of results, incl. fairness, in the context of objectives and prior studies. | Discussion — Principal Results; Comparison with Prior Work. |
| Limitations | **26** | Discuss limitations and their effects on bias, uncertainty, and generalizability. | Discussion — Limitations. |
| Usability | **27a** | Describe how poor-quality or unavailable input data should be handled at implementation. | Discussion — Limitations / future directions. |
|  | **27b** | Discuss whether users must interact with input/model and required expertise. | Discussion — Conclusions. |
|  | **27c** | Discuss next steps for future research, applicability, and generalizability. | Discussion — Conclusions. |

Abbreviations: AUROC, area under the receiver operating characteristic curve; AUPRC, area under the precision-recall curve; ECG, electrocardiogram; EMR, electronic medical record; ICD-10, International Classification of Diseases, 10th revision; KED, Knowledge-Enhanced Diagnosis; NRI, net reclassification improvement; NSR, normal sinus rhythm; PPI, patient and public involvement; YSH, Yongin Severance Hospital.
