# Supplementary material for: Electrocardiogram Embeddings Beyond Chronological Age for Prediagnostic Cognitive Impairment and Dementia: Development and External Validation Study
Source: J Med Internet Res. 2026 Oct 23;28:e108392. doi: 10.2196/108392 (PMC13743964; doi:10.2196/108392)
Supplement: Supplementary file 4 [file jmir_v28i1e108392_app4.docx]

**Multimedia Appendix 4**

**Sensitivity analysis for potential misclassification of control patients (external validation cohort)**

In the external validation cohort, the only site where additional record extraction was permitted, we searched clinical notes and prescription records for evidence of dementia among control patients. Of 5,200 control patients, 35 control patients (0.67%; 60 ECGs) had evidence of unrecorded dementia. The external evaluation was then repeated after excluding these patients. The fitted models and the classification thresholds from the main analysis were held fixed, so that any change is attributable to the labels alone; no model was refitted. Confidence intervals were obtained by patient-level cluster bootstrap with 2,000 resamples. Table S1 reports performance under each scenario, and Table S2 compares the predicted probabilities of flagged and other controls.

**Table S1. Model performance with all controls and after excluding controls with possible unrecorded dementia**

| **Model** | **Controls** | **AUROC** | **AUPRC** | **Sensitivity** | **Specificity** | **PPV** | **NPV** | **F1 score** | **Accuracy** |
| --- | --- | --- | --- | --- | --- | --- | --- | --- | --- |
| **ECG only** | All controls | 0.7735  (0.7571–0.7896) | 0.5137  (0.4839–0.5445) | 0.8276  (0.8061–0.8491) | 0.5506  (0.5367–0.5646) | 0.3452  (0.3257–0.3652) | 0.9177  (0.9059–0.9293) | 0.4872  (0.4667–0.5088) | 0.6122  (0.5998–0.6248) |
|  | Flagged controls excluded | 0.7762  (0.7604–0.7922) | 0.5223  (0.4922–0.5536) | 0.8276  (0.8061–0.8491) | 0.5545  (0.5405–0.5682) | 0.3489  (0.3295–0.3692) | 0.9177  (0.9059–0.9293) | 0.4909  (0.4702–0.5122) | 0.6157  (0.6031–0.6282) |
| **Age only** | All controls | 0.8093  (0.7933–0.8239) | 0.5050  (0.4751–0.5366) | 0.7538  (0.7238–0.7845) | 0.7117  (0.6973–0.7256) | 0.4281  (0.4039–0.4517) | 0.9099  (0.8968–0.9224) | 0.5460  (0.5220–0.5684) | 0.7210  (0.7079–0.7344) |
|  | Flagged controls excluded | 0.8134  (0.7976–0.8279) | 0.5188  (0.4881–0.5512) | 0.7538  (0.7238–0.7845) | 0.7167  (0.7024–0.7305) | 0.4343  (0.4099–0.4584) | 0.9098  (0.8967–0.9224) | 0.5511  (0.5268–0.5741) | 0.7250  (0.7117–0.7384) |
| **ECG probability + age** | All controls | 0.8253  (0.8109–0.8392) | 0.5576  (0.5265–0.5893) | 0.8329  (0.8085–0.8573) | 0.6636  (0.6491–0.6778) | 0.4148  (0.3931–0.4376) | 0.9328  (0.9221–0.9434) | 0.5538  (0.5322–0.5760) | 0.7013  (0.6888–0.7142) |
|  | Flagged controls excluded | 0.8290  (0.8145–0.8432) | 0.5697  (0.5383–0.6028) | 0.8329  (0.8085–0.8573) | 0.6683  (0.6540–0.6826) | 0.4201  (0.3984–0.4425) | 0.9327  (0.9220–0.9433) | 0.5585  (0.5367–0.5810) | 0.7051  (0.6924–0.7181) |

Values are point estimates with 95% CIs, obtained from 2,000 iterations of exact-age rematching combined with patient-level bootstrap resampling.

**Table S2. Predicted probabilities of flagged controls and other controls.**

| **Controls** | **ECGs, n** | **ECG-only** | | **Age-only** | | **ECG probability + age** | |
| --- | --- | --- | --- | --- | --- | --- | --- |
|  |  | **Mean ± SD** | **Median [IQR]** | **Mean ± SD** | **Median [IQR]** | **Mean ± SD** | **Median [IQR]** |
| Flagged controls | 60 | 0.7403 ± 0.1362 | 0.7927  [0.6789–0.8368] | 0.7943 ± 0.1233 | 0.8544  [0.7399–0.8791] | 0.8226 ± 0.1114 | 0.8510  [0.8185–0.8937] |
| Other controls | 7,862 | 0.4498 ± 0.2415 | 0.4493  [0.2195–0.6701] | 0.4249 ± 0.2222 | 0.4052  [0.2467–0.5898] | 0.4133 ± 0.2548 | 0.3782  [0.1810–0.6344] |

*Abbreviations:* AUPRC, area under the precision–recall curve; AUROC, area under the receiver operating characteristic curve; CI, confidence interval; ECG, electrocardiogram; IQR, interquartile range; NPV, negative predictive value; PPV, positive predictive value; SD, standard deviation.
