# Supplementary material for: Electrocardiogram Embeddings Beyond Chronological Age for Prediagnostic Cognitive Impairment and Dementia: Development and External Validation Study
Source: J Med Internet Res. 2026 Oct 23;28:e108392. doi: 10.2196/108392 (PMC13743964; doi:10.2196/108392)
Supplement: Supplementary file 5 [file jmir_v28i1e108392_app5.docx]

**Multimedia Appendix 5**

**Model performance in the exact-age 1:1 matched case–control set**

Because age is the dominant predictor in this setting and matching on each individual's median age left a residual imbalance at the ECG level, all three models were additionally evaluated in an exact-age 1:1 matched case–control set. Within each integer year of age at ECG acquisition, cases and controls were matched 1:1 without replacement, so that the age distributions of the two groups are identical by construction and age alone is uninformative. No model was refitted and the classification thresholds from the main analysis were applied unchanged; only the evaluation set differed. Because a single matched set depends on which individuals are sampled, matching was repeated and combined with bootstrap resampling of the matched set (2,000 iterations in total), so that the 95% CIs reflect both sources of variability.

**Table S1. Discriminative performance of the three models in the exact-age 1:1 matched case–control set**

| **Cohort / model** | **AUROC** | **AUPRC** | **Accuracy** | **Sensitivity** | **Specificity** | **PPV** | **NPV** | **F1 score** |
| --- | --- | --- | --- | --- | --- | --- | --- | --- |
| **Internal validation (Severance)** | | | | | | | | |
| Age only | 0.5005  (0.4876–0.5138) | 0.5014  (0.4903–0.5132) | 0.5004  (0.4893–0.5119) | 0.5912  (0.5759–0.6075) | 0.4096  (0.3938–0.4257) | 0.5004  (0.4909–0.5101) | 0.5005  (0.4869–0.5146) | 0.5420  (0.5307–0.5534) |
| ECG only | 0.6324  (0.6191–0.6461) | 0.6520  (0.6383–0.6662) | 0.5993  (0.5878–0.6118) | 0.6767  (0.6612–0.6928) | 0.5219  (0.5040–0.5396) | 0.5860  (0.5757–0.5968) | 0.6175  (0.6036–0.6315) | 0.6281  (0.6167–0.6392) |
| ECG probability + age | 0.5539  (0.5406–0.5674) | 0.5427  (0.5305–0.5559) | 0.5400  (0.5286–0.5519) | 0.6447  (0.6294–0.6604) | 0.4353  (0.4187–0.4524) | 0.5331  (0.5236–0.5430) | 0.5506  (0.5361–0.5657) | 0.5836  (0.5725–0.5944) |
| **External validation (YSH)** | | | | | | | | |
| Age only | 0.5000  (0.4820–0.5179) | 0.5007  (0.4858–0.5160) | 0.4999  (0.4869–0.5134) | 0.7459  (0.7267–0.7643) | 0.2538  (0.2357–0.2723) | 0.4999  (0.4912–0.5090) | 0.4998  (0.4738–0.5262) | 0.5986  (0.5871–0.6102) |
| ECG only | 0.5775  (0.5581–0.5971) | 0.5939  (0.5753–0.6134) | 0.5252  (0.5111–0.5399) | 0.8067  (0.7895–0.8240) | 0.2436  (0.2213–0.2666) | 0.5161  (0.5070–0.5256) | 0.5575  (0.5251–0.5906) | 0.6295  (0.6185–0.6404) |
| ECG probability + age | 0.5390  (0.5209–0.5577) | 0.5362  (0.5192–0.5547) | 0.5225  (0.5087–0.5365) | 0.8064  (0.7895–0.8240) | 0.2386  (0.2177–0.2604) | 0.5144  (0.5056–0.5233) | 0.5520  (0.5207–0.5831) | 0.6281  (0.6173–0.6386) |

Values are point estimates with 95% CIs, obtained from 2,000 iterations of exact-age rematching combined with patient-level bootstrap resampling.

Age alone is uninformative in this set by construction; the age-only model therefore performs at chance level, and the fusion model, which combines the ECG-derived probability with age, performs below the ECG-only model.

Threshold-dependent metrics (accuracy, sensitivity, specificity, PPV, NPV, and F1 score) are reported for completeness only. The classification thresholds were carried over unchanged from the main analysis, whereas the matched set has a case prevalence of 50%; these metrics are therefore not comparable with those of the main analysis.

*Abbreviations:* AUPRC, area under the precision–recall curve; AUROC, area under the receiver operating characteristic curve; CI, confidence interval; ECG, electrocardiogram; NPV, negative predictive value; PPV, positive predictive value; YSH, Yongin Severance Hospital.
