# Supplementary material for: Electrocardiogram Embeddings Beyond Chronological Age for Prediagnostic Cognitive Impairment and Dementia: Development and External Validation Study
Source: J Med Internet Res. 2026 Oct 23;28:e108392. doi: 10.2196/108392 (PMC13743964; doi:10.2196/108392)
Supplement: Supplementary file 6 [file jmir_v28i1e108392_app6.docx]

**Multimedia Appendix 6**

**Pre-diagnostic trajectories of ECG interpretation labels in the exact-age matched case–control set**

On the exact-age (1:1) matched case–control set, each of the 105 KED interpretation-label probabilities was tested for a group-by-time interaction (a widening case–control difference over the pre-diagnostic period). Because a patient could contribute multiple ECGs, the interaction was estimated with patient-clustered robust standard errors. The 19 labels below were retained (Benjamini–Hochberg *q* < .05 for the interaction and a widening case–control gap toward diagnosis; see Methods) and are ordered by the imminent-window (0–1 year) effect size. Cohen *d* is the standardized case–control difference on the log-probability scale (positive = higher in cases); each column reports Cohen *d* within one pre-diagnostic window.

**Table S1. Window-specific case-control differences (Cohen’s d) for the 19 qualifying ECG interpretation labels in the exact-age matched case-control set**

| **ECG interpretation label** | **Interaction**  **q** | **Remote**  **(5–10 y)** | **Intermediate**  **(3–5 y)** | **Proximal**  **(1–3 y)** | **Imminent**  **(0–1 y)** |
| --- | --- | --- | --- | --- | --- |
| Non-specific lateral ST segment changes | .003 | +0.07 | +0.04 | **+0.16*** | **+0.25*** |
| Non-specific inferior lateral T wave changes | .003 | +0.09 | +0.10 | **+0.17*** | **+0.24*** |
| Non-specific inferior T wave changes | .048 | +0.10 | +0.07 | **+0.16*** | **+0.24*** |
| Non-specific inferior and lateral T wave changes | .040 | +0.05 | +0.01 | **+0.14*** | **+0.22*** |
| Acute myocardial infarction | .012 | +0.06 | −0.02 | **+0.13*** | **+0.21*** |
| Accelerated junctional rhythm | .040 | +0.13 | +0.13 | **+0.14*** | **+0.21*** |
| Non-specific septal T wave changes | .010 | +0.01 | −0.03 | **+0.11*** | **+0.21*** |
| Atrial flutter | .040 | +0.05 | +0.08 | **+0.09*** | **+0.21*** |
| Acute ST segment elevation myocardial infarction | .037 | +0.06 | −0.02 | **+0.13*** | **+0.20*** |
| Non-specific lateral ST T changes | .040 | +0.07 | +0.04 | **+0.13*** | **+0.18*** |
| Normal ECG except for rate | .006 | +0.02 | −0.00 | **+0.11*** | **+0.18*** |
| Non-specific inferior lateral ST T changes | .020 | +0.05 | +0.05 | **+0.11*** | **+0.18*** |
| Abnormal ECG | .005 | −0.00 | −0.04 | **+0.10*** | **+0.17*** |
| Non-specific ST segment junctional depression | .003 | +0.02 | −0.02 | **+0.10*** | **+0.16*** |
| Early repolarization | .013 | +0.02 | −0.07 | **+0.08*** | **+0.16*** |
| Borderline ECG | <.001 | −0.00 | −0.03 | **+0.10*** | **+0.16*** |
| Ectopic atrial rhythm | <.001 | +0.01 | +0.01 | +0.06 | **+0.11*** |
| Left atrial abnormality | .029 | −0.02 | +0.01 | +0.06 | **+0.09*** |
| Right atrial abnormality | .023 | −0.07 | −0.08 | −0.00 | +0.01 |

Cohen *d* values marked with an asterisk (*) and shown in bold are significant at Benjamini–Hochberg *q* < .05 within that window (correction applied across labels within each window, based on the patient-clustered adjusted *P* value).

Interaction *q* = Benjamini–Hochberg–corrected *P* value for the group-by-time interaction (least-squares regression on log-probability with patient-clustered robust standard errors), corrected across the 105 labels.

*Abbreviations:* ECG, electrocardiogram; KED, Knowledge-Enhanced Diagnosis; ST, ST segment.

**Table S2. Number of ECGs per patient by pre-diagnostic window in the exact-age matched case-control set**

| Window | ECGs per patients in Case | ECGs per patients in Control |
| --- | --- | --- |
| Remote (5-10y) | 1.81 | 1.11 |
| Intermediate (3-5y) | 1.60 | 1.11 |
| Proximal (1-3y) | 1.74 | 1.16 |
| Imminent (0-1y) | 1.75 | 1.23 |

*Abbreviations:* ECG, electrocardiogram
