# Supplementary material for: Electrocardiogram Embeddings Beyond Chronological Age for Prediagnostic Cognitive Impairment and Dementia: Development and External Validation Study
Source: J Med Internet Res. 2026 Oct 23;28:e108392. doi: 10.2196/108392 (PMC13743964; doi:10.2196/108392)
Supplement: Supplementary file 7 [file jmir_v28i1e108392_app7.docx]

**Multimedia Appendix 7**

Assessment of Temporal Bias and Proximity-to-Diagnosis Effects in the External Validation Cohort

To assess whether the overall performance was driven by ECGs recorded shortly before diagnosis, we examined the external validation cohort (Yongin Severance Hospital) using the predicted probabilities of the final models without retraining. ECGs were assigned to four pre-diagnostic windows according to the interval between ECG acquisition and the index date: remote (5–10 years), intermediate (3–5 years), proximal (1–3 years), and imminent (0–1 year).

To remove the effect of the uneven distribution of ECGs across windows, we compared cases and controls within the same window and computed the AUROC of the age-only, ECG-only, and fusion (ECG probability + age) models in each window. 95% CIs were estimated by patient-level cluster bootstrap resampling with 2000 iterations.

**Table S1. Model discrimination with cases and controls compared within the same pre-diagnostic window (external validation cohort).**

| **Window** | **No. of ECGs** | | **AUROC (95% CI)** | | |
| --- | --- | --- | --- | --- | --- |
|  | **Cases** | **Controls** | **Age only** | **ECG only** | **ECG probability + age** |
| Remote (5–10 y) | 320 | 383 | 0.804 (0.762–0.844) | 0.762 (0.719–0.802) | 0.822 (0.784–0.857) |
| Intermediate (3–5 y) | 315 | 478 | 0.824 (0.785–0.859) | 0.766 (0.722–0.808) | 0.837 (0.799–0.873) |
| Proximal (1–3 y) | 514 | 961 | 0.835 (0.807–0.861) | 0.784 (0.755–0.813) | 0.844 (0.818–0.869) |
| Imminent (0–1 y) | 1,115 | 6,100 | 0.822 (0.802–0.840) | 0.786 (0.767–0.806) | 0.838 (0.820–0.856) |

*Abbreviations*: AUROC, area under the receiver operating characteristic curve; CI, confidence interval.
